# Association of the genetic variants of luteinizing hormone, luteinizing hormone receptor and polycystic ovary syndrome

**DOI:** 10.1186/1477-7827-10-36

**Published:** 2012-04-30

**Authors:** Nana Liu, Yanmin Ma, Shuyu Wang, Xiaowei Zhang, Qiufang Zhang, Xue Zhang, Li Fu, Jie Qiao

**Affiliations:** 1 Center for Reproductive Medicine, Department of Obstetrics and Gynecology, Peking University Third Hospital, Beijing, 100191, Peoples Republic of China; 2 Key Laboratory of Assisted Reproduction, Ministry of Education, Beijing, 100191, China; 3 Beijing Key Laboratory of Reproductive Endocrinology and Assisted Reproduction, Beijing, 100191, China; 4 Reproductive Medical Centre, Beijing Obstetrics and Gynecology Hospital affiliated of Capital Medical University, Beijing, 100026, China

**Keywords:** Luteinizing hormone, Luteinizing hormone receptor, Polycystic ovary syndrome, Gene polymorphism

## Abstract

**Background:**

High circulating luteinizing hormone (LH) level is a typical biochemical feature of polycystic ovary syndrome (PCOS) whose pathophysiology is still unclear. Certain mutations of LH and LH receptor (LHR) may lead to changes in bioactivity of these hormones. The aim of this study was determine the role of the LH and LHR polymorphisms in the pathogenesis of PCOS using a genetic approach.

**Methods:**

315 PCOS women and 212 controls were screened for the gene variants of *LH* G1052A and *LHR* rs61996318 polymorphisms by polymerase chain reaction restriction fragment length polymorphism (PCR-RFLP).

**Results:**

PCOS patients had significantly more A allele frequency of *LH* G1052A mutations than controls (p=0.001). Within PCOS group, carriers of *LH* 1052A allele had lower LH (p=0.05) and higher fasting glucose levels (p=0.04). No subjects were identified with *LHR* rs61996318 polymorphisms. A new *LHR* single nucleotide polymorphism (SNP) was found without clear association with PCOS.

**Conclusions:**

Results suggested *LH* G1052A mutation might influence PCOS susceptibility and phenotypes.

## Background

PCOS is one of the most common endocrine disorders in women of reproductive age, and it affects about 1 in 15 women worldwide [1]. The syndrome is characterized by chronic anovulation or infrequent ovulation, hyperandrogenism and numerous follicular cysts in enlarge ovaries. Patients with PCOS are susceptible to infertility, obesity and insulin resistance [1]. Despite extensive research, the precise etiology and mechanisms of PCOS remain largely unknown. Considerable interest in the genetics of PCOS has increased in recent years following increasing number of familial aggregation studies. These studies have demonstrated genetic component of PCOS by the increasing risk ratio of siblings of PCOS individuals compared with that of the general population [2,3].

One typical biochemical feature of PCOS is the high circulating LH level which is thought to be associated with the abnormal patterns of gonadotropin pulsatility in pituitary [4]. LH is a member of the glycoprotein hormone family that also includes follicle stimulating hormone (FSH), thyroid-stimulating hormone and human chorionic gonadotropin. These hormones are : heterodimers in which the -subunit is common to all hormones and the -subunit is unique and confers biologic specificity [5,6]. The effect of LH is mediated by LHR which is expressed in the theca cells and granulose cells. Abnormal LH signaling is believed to play a permissive role in augmenting ovarian androgen production in PCOS and increasing the likelihood of anovulation [1,7].

Studies have shown that *LH* and *LHR* gene mutations may change the structure or function of the LH and LHR, either activating or inactivating their bioactivity, which cause anovulation, amenorrhea and polycystic ovary in women [8][10]. There are compelling evidences for the genetic determinism of *LH and LHR* for PCOS, although the results of different populations and loci of mutation showed inconsistencies. Two missense point mutations in the *LH* gene (Trp 8 Arg and Ile 15 Thr) were reported to associate with PCOS in Japan and obese PCOS women in UK. But a study of obese PCOS from north European found *LH* gene (Trp 8 Arg and Ile 15 Thr) were in lower frequency [11][13]. Another *LH* gene variant G1502A in exon 3 (Gly102Ser) was found to be higher in Singapore Chinese women who had menstrual disorders [14]. Yet a Korean research found no difference of *LH* gene Gly102Ser in PCOS patients [15]. Women with *LHR* mutations often show amenorrhea and infertility [5]. Chen et al. had conducted genome-wide association study (GWAS) of PCOS in Han Chinese women and found strong evidence of associations between PCOS and *LHR* gene loci [16]. PCOS had lower frequency of *LHR 18insLQ* genotype compared with controls (24.9% vs 28%) in a study which there was about 15% lower risk for PCOS per minor allele copy, though the lower LHR 18insLQ frequency was not significant after Bonferronis correction between PCOS and controls [17]. Given the above evidences, we decide to study both *LH* and *LHR* gene variants in PCOS patients of Chinese Han women, intending to further elucidate the genetic mutation mechanism of *LH* and *LHR* with PCOS.

## Methods

### Patients

315 Chinese Han women with PCOS and 212 controls were recruited at Peking University Third Hospital Reproductive Centre from January 2010 to March 2012. This study was approved by the institutional ethics committee of the Hospital, and written informed consent was obtained from each subject.

PCOS was diagnosed according to the 2003 Rotterdam criteria [18]. Two out of three of the following criteria were met for the diagnosis: oligo-ovulation and/or anovulation,irregular menstrual cycle ( less than eight periods per year, or cycles that are longer than 35days, and amenorrhoea is absence of menstruation for more than 3months without pregnancy), clinical and/or biochemical signs of hyperandrogenism (Ferriman-Gallwey score6 or total serum testosterone>2.8nmo/l ) and polycystic ovaries (PCO) by transvaginal ultrasound (12 or more follicles in each ovary measuring 29mm in diameter, and/or increased ovarian volume, >10ml). Patients with the following disorders were excluded: hyperprolactinemia, nonclassic congenital adrenal hyperplasia, Cushings syndrome, androgen secreting neoplasm and thyroid dysfunction. The control group included age matched women with regular menstrual cycles. They were infertile women for tubal obstruction and/or male factor. They had no clinical or biochemical signs of hyperandrogenism and no polycystic ovaries. All subjects (range from 20 to 40years old) had not taken hormonal therapy (including oral contraceptives) for at least 3months prior to testing.

### Hormonal and biochemical measures

We collected fasting blood samples of all subjects during the follicular phase of a menstrual cycle (spontaneous or bleeding after progestin withdrawal). Serum LH, FSH, testosterone (T), androstenedione (A) and insulin concentrations were determined by chemiluminescence immunoassay. Glucose levels, total cholesterol (CHO) and triglycerides (TG) were determined by oxidase methods. High-density lipoprotein (HDL) by synthetic polymer/detergent HDL-C assay and low-density lipoprotein (LDL) by surfactant LDL-C assay. Body mass index (BMI) was calculated as body weight (kg) divided by body height squared (m^2^). Waist hip ratio (WHR) was calculated as hipline divided by waistline.

### Genotyping

Genomic DNA was extracted from peripheral blood leukocytes using the standard salting out method [19]. The purity and concentration of the isolated DNA was measured. Primers of *LH* G1502A forward: AGTCTGAGACCTGTGGGGTCAGCTT, reverse: GGAGGATCCGGGTGTCAGGGCTCCA. Primers of *LHR* rs61996318: forward: TGATGGTGGTGGTGATGATG, reverse: GGTTTCTAGCCA GCCAGTTG. PCR amplification was performed as Nam Keun Kim et al. described [15]. The fragment of *LHR* rs61996318 amplification is 379bp. PCR products were analyzed by RFLP using Enzyme Rsal and then they were given to electrophoresis on a 2% agarose gel stained with ethidium bromide. In Genebank, *LHR* rs61996318 mutation is from GA, and the LHR protein of corresponding position changes from Gln to Lys.

### DNA sequencing

The *LHR* PCR products (315 PCOS and 122 controls) were sequenced in both directions using primers matching intronic boundaries (primer sequences as mentioned before). All were done by automatic genotype sequencing instrument (ABI3730XL,BigdyeV3.1) in Peking Huada Gene Company.

### Statistical analysis

Normal distribution of all clinical parameters was analyzed using the Kolmogorov-Smirnov test. Comparisons of normal distribution continuous variables between groups were independent studentst test. When continuous variables were not normal distribution, MannWhitney U-test was used. Fishers exact test and chi-square test was used for analyzing the associations between categorical variables. All analyses were performed by SPSS software version 18.0 (SPSS Inc. Chicago, IL, USA). NC-pass was used to perform power calculation. Tests of statistical significance were two sided and taken as significant when p 0.05.

## Results

### Clinical parameters

The basal demographical, hormonal and biochemical parameters of controls and PCOS women are summarized in Table 1. The age and WHR between two groups were similar. Similar with most studies, LH and testosterone levels were higher in PCOS patients compared with that of controls, the same as BMI, LH/FSH ratio, A and TG levels. FSH level was significantly lower in PCOS. There were no significant difference in the levels of fasting glucose, fasting insulin, HDL, LDL and CHO between PCOS patients and controls (Table 1).

**Table 1:** Clinical and endocrine-metabolic parameters in PCOS and control women

Parameters	PCOS(N=315)	Controls(N=212)
Age(yr)	32.483.88	33.024.26
BMI(kg/m2) WHR	24.183.910.830.07	22.433.57*0.830.06
Oligo- or anovulation (%)	91.42	0*
PCO (%)	93	0*
Testosterone ( nmol/L)	1.801.10	0.910.44*
A( nmol/L)	13.665.56	6.352.16*
LH(mIU/ml)	9.096.42	4.202.69*
FSH(mIU/ml) LH/FSH	6.191.941.500.99	7.312.09*0.610.43*
Fasting glucose (mmol/L)Fasting insulin(uIU/ml)CHO(mmol/L)	4.940.7013.099.294.870.87	5.070.8812.928.634.680.81
TG(mmol/L)HDL(mmol/L)LDL(mmol/L)	1.551.001.260.312.991.44	0.930.48*1.310.252.760.84

Independent studentst test and MannWhitney U-test; Data are described in meanSD; BMI: body mass index; WHR: waist hip ratio; PCO: polycystic ovary syndrome; A: serum androstenedion; CHO: total cholesterol; TG: triglycerides; HDL: High-density lipoprotein; LDL:low-density lipoprotein. *: P0.05.

### Variants of *LH* and *LHR* genes polymorphisms

In PCOS group, there are 3 *LH* A1052A homozygous patients, 10 *LH* G1052A heterozygous patients, and the gene variants frequencies were 1.0% and 3.2% respectively. There was no one *LH G*1052A gene variant in 212 controls. The *LH* G1052A heterozygous frequency in PCOS was significantly higher than that in controls (p=0.007) and A allele frequency with PCOS patients was more than that in controls (p=0.001, Table 2).

**Table 2:** ***LH*G1052A genotype distribution in PCOS and control women**

LH genotype	PCOS(N=315)	Controls(N=212)	P
G1052G	302(95.8%)	212(100%)	-
G1052A	10(3.2%)	0(0%)	0.007
A1052A	3(1.0%)	0(0%)	0.27
Alleles	-	-	-
G	614(97.5%)	414(100%)	-
A	16(2.5%)	0(0%)	0.001

Fishers exact test; Data are presented as number (percentage). ()=not tested.

There were all wild type of *LHR* rs61996318 SNP polymorphisms in PCOS and control subjects. In order to check its accuracy, the PCR products of 315 PCOS and 122 controls were subjected to genetic DNA sequencing and confirmed the same result with PCR-RFLP.

### A new SNP in *LHR* gene

In the *LHR* PCR products DNA sequencing map, we found a new SNP in *LHR* gene NT27762960 position near exon 7, where there was a loss of base A, in which the gene sequencing changes from TAGCC A GAG to TAGCC_GAG.

In PCOS group, there were 2 homozygous of this new SNP and in controls there were 5 (p=0.02). But the heterozygous frequency with PCOS was higher than that in controls (21.3% vs. 13.9%, p=0.11). There was no significant difference of missing base frequency in the two groups (11.3% vs. 11.1%, Table 3).

**Table 3:** ***LHR*new SNP genotype distribution in PCOS and control women**

*LHR* new SNP genotype	PCOS(N=315)	Controls(N=122)	P
wild type	246(78.1%)	100(82.0%)	-
homozygous type	2(0.6%)	5(4.1%)	0.03
heterozygous type	67(21.3%)	17(13.9%)	0.11
Alleles	-	-	-
A	559(88.7%)	217(88.9%)	-
Missing	71(11.3%)	27(11.1%)	0.93

Chi-square test; Data are presented as number and (percentage). ()=not tested.

### PCOS phenotype by different *LH* and *LHR* genotypes

Within the PCOS cohort, genotypes of the *LH* G1052A mutations were not associated with general clinical signs, such as BMI, waist hip ratio, amenorrhea and PCO (Table 4). Carriers of the *LH* 1052A allele showed lower LH level (p=0.05), higher fasting glucose level (p=0.04) and the power calculation was 79% and 14% respectively. Although the testosterone and androstenedione levels were similar between two *LH* genotypes groups, the testosterone levels were 2.35, 2.58 and 4.09nmol/L with three *LH* A1052A homozygous PCOS subjects respectively (data wasnt shown in Table 4). FSH, TG, fasting insulin levels and LH FSH ratio were similar between two groups.

**Table 4:** **Clinical and endocrine-metabolic parameters with different*LH*G1052A genotype and*LHR*new SNP genotype in PCOS patients**

	*LH*1052G/G (N=302)	*LH*1052G/A+A/A (N=13)	*LHR* wild type (N=246)	*LHR* mutated type (N=69)
BMI(kg/m2)	24.243.93	22.743.83	24.203.91	24.154.03
WHR	0.830.07	0.810.05	0.830.07	0.820.06
Amenorrhea,n(%)	276(91.39)	11(84.62)	223(90.65)	65(94.20)
PCO,n(%)	282(93.37)	11(84.62)	227(92.28)	66(95.65)
LH(mIU/ml)	9.236.44	5.714.07*	8.856.47	9.966.06
FSH(mIU/ml)	6.211.91	5.902.28	6.191.97	6.231.82
LH/FSH	1.520.99	1.091.06	1.471.02	1.610.88
Testosterone ( nmol/L)	1.801.10	1.911.04	1.791.07	1.861.22
A( nmol/L)	13.665.59	13.764.59	13.675.50	13.635.78
Fasting glucose(mmol/L)	4.920.63	5.321.54*	4.930.73	4.980.60
Fasting insulin(uIU/ml)	9.706.30	13.279.38	12.839.13	14.249.88
TG(mmol/L)	1.561.01	1.260.49	1.550.97	1.551.07

Independent studentst test and MannWhitney U-test; Data are described in meanSD. P0.05 was considered statistically significant. *: P0.05.

For patients with new SNP of *LHR* gene in PCOS group, the LH, fasting glucose and testosterone levels didnt differ between mutated type and wild type. No association of the new SNP genotypes with BMI, WHR, amenorrhea or PCO was observed. FSH, LH/FSH, TG and fasting insulin levels were also similar between two groups (Table 4).

**Figure 1:**
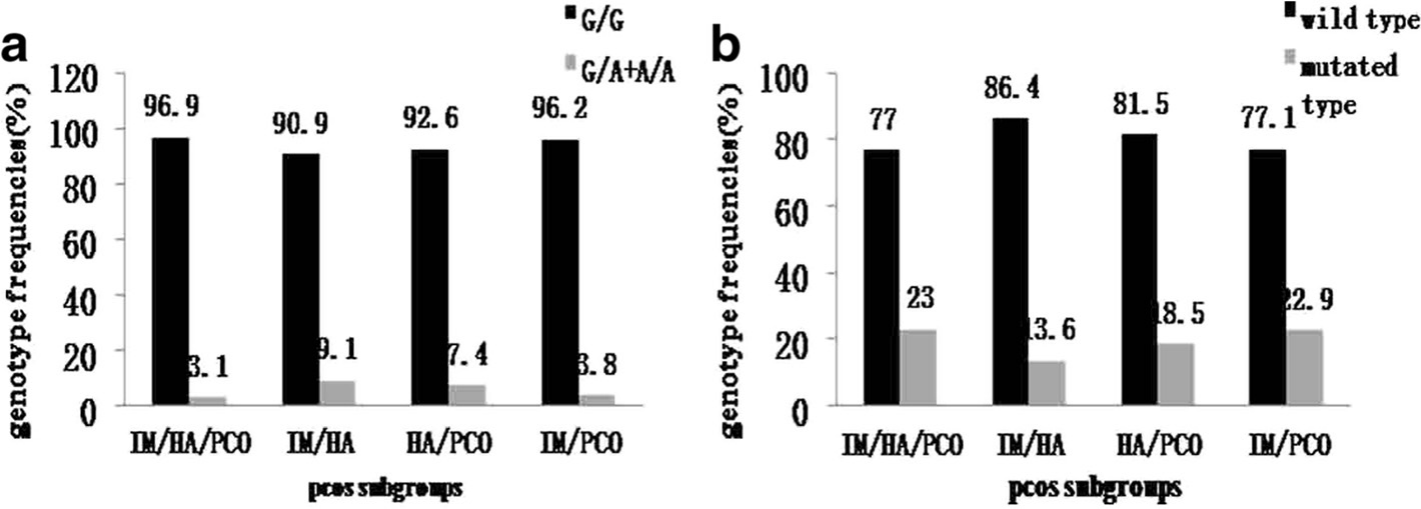
**Distribution of*LH*G1052A genotype (a) and*LHR*new SNP genotype (b) in four subgroups of PCOS.** PCOS patients were divided into four subgroups according to 2003 Rotterdam criteria (IMA/HA/PCO, IM/HA, HA/PCO and IM/PCO). There were no significant differences of these two gene mutations among four subgroups of PCOS (*LH* G1052A genotype: p=0.46; *LHR* new SNP genotype: p=0.74).

As mentioned above, symptoms or signs of PCOS include irregular menses (IM), clinical or biochemical hyperandrogenism (HA) and PCO according to 2003 Rotterdam criteria. There are four possible diagnostic subcategories of PCOS *i.e.* IM/HA/PCO, IM/HA, HA/PCO, and IM/PCO. We analyzed and compared the distribution of *LH* G1052A genotype and *LHR* new SNP genotype according to these four subgroups of our subject pool (Figure 1). The *LH* G1052A gene variants frequency was 9.1% in IM/HA subgroup and 7.4% in HA/PCO subgroup. Both are slightly higher than that of IM/HA/PCO subgroup and IM/PCO subgroups (3.1% and 3.8%). But the difference of A allele frequency was not significant among four subgroups (p=0.46). The four PCOS subgroups showed similar gene variants frequency of the *LHR* new SNP. IM/HA/PCO subgroup was 23%; IM/HA subgroup was 13.60%; HA/PCO was 18.5% and IM/PCO was 22.9% (p=0.74).

## Discussion

Until now, the relationship between LH signaling pathway and PCOS has not been defined clearly. Besides the two-cell-two-gonadotrophin theory, LH plays a critical role in the folliculogenesis. During the second half of the follicular phase, LH regulates mRNA concentrations in granulose cells for numerous genes that function in autocrine and paracrine signaling which can help the follicle developing well [20,21]. LH promotes the secretion of androgens by ovarian theca cells, which may result in follicular maturation arrest [22]. LHR is over expressed in theca cells and granulose cell from PCOS patients [23,24]. Discovery of SNP as new markers of the human genome opened novel ways to demonstrate genetic associations of candidate genes to complex disorders. A number of functional SNPs have been described in *LH* and *LHR* genes which may affect the LH and LHR bioactivity [11,25]. In the current study, we investigated both the genotype of *LH* G1052A and *LHR* rs61996318 in Han Chinese PCOS patients. *LH* 1052A allele frequency in PCOS group was higher than that of controls (p=0.001).Within PCOS group, *LH*1052A gene variants showed influence on LH level (p=0.05) and fasting glucose level (p=0.04). No *LHR* rs61996318 mutation was found in our subjects. For the first time, we found a new SNP in *LHR* gene NT27762960 where a base A was missing. To our knowledge, this is the first study to investigate the correlations of both *LH* and *LHR* genotypes in PCOS.

In our study, the frequencies of *LH* G1052A homozygous and heterozygous variants were 1.0% (3/315) and 3.2% (10/315) respectively in PCOS. In contrast, there was no *LH* G1052A mutation (0/212) in control group. The frequency of A allele in PCOS was significantly higher than controls (p=0.001). Similar with our result that the *LH* G1052A frequency was about 4.2%, Ramanujam et al. also reported a *LH* G1052A variant frequency of 4% (7/176) in Singapore Chinese women with menstrual disorders and no one A allele in the control subjects (0/200). They speculated *LH* G1052A mutations affected gonadal function, and the micro heterogeneity was related to menstrual irregularity [14]. But Kim et al. studied 108 Korean women patients with endometriosis or PCOS and found none of them were *LH* G1052A homozygous gene variants [15]. Ramanujam et al. didnt find any variant either in Malays or Indians PCOS women [26]. These results suggested that ethnicity and environment may influence the genetic variability in PCOS patients. Specifically, different ethnical and regional group may express different *LH* G1052A genotype.

*LH* G1052A mutation is a single missense mutation in exon 3 of the *LH* gene which replaces glycine with serine at amino acid 102 [13]. Haavisto et al. had uncovered another LH mutation (Trp8Arg and Ile15Thr, V-LH) which alters the biological function of LH with an elevation of in vitro bioactivity compared to that of wild type LH. Heterozygous women for the V-LH allele had higher levels of serum testosterone, and estradiol [12,27]. Within PCOS group, we found that carriers of the *LH* 1052A allele had lower LH level (5.71 vs 9.23 mIU/ml, p=0.05). The power calculation to LH level between two *LH* G1052A genotypes within PCOS was 79%. Additionally, testosterone levels of three *LH* A1052A homozygous carriers were 2.35, 2.58 and 4.09nmol/L (data were not shown in table) respectively. It has been well known that LH plays an important role in secreting testosterone. This phenomenon strongly suggested *LH* G1052A gene variants had an effect on LH protein and may enhance the LH bioactivity. Lamminen et al. had found that *LH* G1052A mutation may not affect receptor binding and bioactivity of LH [28]. However, PCOS is a heterogeneous endocrine disorder, which is involved in many factors, such as genetics and environment factors. Thus, the biological function of *LH* 1052A gene variants within PCOS microenvironment needs to be further studied.

Carriers of the *LH* 1052A allele had higher fasting glucose level (5.32 vs 4.92mmol/l, p=0.04). Higher fasting glucose usually represents insulin resistance or metabolic dysfunction, which is one of the most common clinical features of PCOS. Studies have confirmed insulin can enhance the responsiveness of theca cell and granulose cell to LH in PCOS women [24]. Insulin resistance and LH interacted with each other and increase the severity of PCOS.

We found a new SNP in *LHR* gene NT27762960 position near exon 7 where base A was missing. The amino acid sequence of *LHR* encoded by the exons 210 has been shown to confer hormone specificity and binding to the gonadotropin [29]. Homozygous or compound heterozygous inactivating gene variants of the *LHR* cause gonadal resistance to hCG and LH, and the latter event causes feedback to pituitary to result in the rising LH level. However, the clinical phenotypes associated with these mutations are closely correlated with the severity of the mutation [30]. Piersma et al., found that *LHR* 18insLQ mutation, Asn291Ser and Ser312Asn render the LHR bioactivity, which resulted LHR was insensitive to LH [25,31]. Valkenburg et al. found in Caucasian PCOS that the *LHR* 18insLQ frequency was lower in PCOS patients and about 15% lower risk for PCOS per minor allele copy. However, Asn291Ser and Ser312Asn were not obviously associated with PCOS. In their study the 18insLQ homozygous carriers showed 24.1% lower levels of estrogen (179 vs 236pmol/l) [17]. In our study, the missing A allele frequency was similar between PCOS and controls (11.3% vs. 11.1%, p=0.93). These LHR new polymorphisms were in HardyWeinberg equilibrium (HWE) within the PCOS population and controls. Within PCOS group, LH level of new *LHR* SNP was a little higher than wild type (9.96 vs. 8.85mIU/ml), but the difference wasnt significant, and the same as levels of testosterone and androstenedione. We also didnt find the relationship between new *LHR* gene SNP and other PCOS phenotypes like PCO, BMI, fasting glucose, and fasting insulin levels. We didnt find the association between the new SNP of *LHR* gene with PCOS susceptibility and phenotype. Further study in this aspect is needed.

High LH level is a significant character of PCOS. Although LH level was no longer as a diagnostic factor of PCOS in 2003 Rotterdam criteria, LH level or LH FSH ratio still acts as a diagnostic criterion of PCOS in some Asian countries such as Japan[32]. Therefore, we analyzed these two gene variants frequencies within four subgroups of PCOS. The minor alleles frequencies of *LH* G1052A genotype and *LHR* new SNP genotype werent significantly different among four subgroups. This result showed that these *LH* G1052A genotype and *LHR* new SNP genotype had little relationship with PCOS diagnosis.

Based on this study, we had some limitations, such as: 1) the number of homozygous and heterozygous with *LH* and *LHR* mutation was small; 2) *LH* G1052A mutation was not in HWE; 3) The power calculation to fasting glucose level between two *LH* G1052A genotypes within PCOS group was low. We thought this phenomenon was related to the low *LH* G1052A mutation frequency, which was also testified in three other studies [14,15,26].

## Conclusions

In summary, the current study provides new insight into the role of LH genetics in the pathophysiology of PCOS. We found the minor allele frequency of *LH* G1052A was higher with PCOS; PCOS carriers of *LH* 1052A allele had lower LH level and high fasting glucose level. We speculated *LH* G1052A mutation may contribute to the pathogenesis of PCOS. The *LHR* rs61996318 SNP maybe highly conserved in Chinese Han women. We also found a new *LHR* SNP for the first time in PCOS and further study is in process to elucidate the associations between this new *LHR* SNP and PCOS.

## Competing interests

All of the authors declare that they have no competing interests.

## Authors' contributions

NL and YM participated in the design of the study, carried out the experiment, performed the statistical analysis and drafted the manuscript. SW, XZ and QZ were involved in revision of manuscript drafts. LF and XZ participated in collection of blood samples. JQ contributed to the design of the experiment and was responsible for finalizing manuscript. All authors read and approved the final manuscript.
